# Association between cannabis use and blood pressure levels according to comorbidities and socioeconomic status

**DOI:** 10.1038/s41598-022-22841-6

**Published:** 2023-02-05

**Authors:** Alexandre Vallée

**Affiliations:** Department of Epidemiology-Data-Biostatistics, Delegation of Clinical Research and Innovation (DRCI), Foch Hospital, 92150 Suresnes, France

**Keywords:** Cardiology, Risk factors

## Abstract

The associations between blood pressure and cannabis use remain inconsistent. The purpose of our study was to examine gender stratified associations of cannabis use and blood pressure [systolic, diastolic blood pressure (BP), pulse pressure (PP)] levels among the general UK Biobank population based study. Among 91,161 volunteers of the UK Biobank population, cannabis use status was assessed by questionnaire and range as heavy, moderate, low and never users. Associations between cannabis use and BP were estimated using multiple gender linear regressions. In adjusted covariates models, lifetime heavy cannabis use was associated with decrease in both SBP, DBP and PP in both genders, but with a higher effect among women (for SBP in men, b = − 1.09 (0.27), p < 0.001; in women, b = − 1.85 (0.36), p < 0.001; for DBP in men, b = − 0.50 (0.15), p < 0.001; in women, b = − 0.87 (0.17), p < 0.001; and for PP in men, b = − 0.60 (0.20), p < 0.001; in women, b = − 0.97 (0.27), p < 0.001. Among men, lower SBP and DBP levels were observed with participants without dyslipidemia and lower PP in participants with high income levels. Among women, lower SBP, DBP and PP were observed with current smokers, moderate/low alcohol levels and participants without dyslipidemia. Current cannabis use was associated with lower SBP levels in men (b = − 0.63 (0.25), p = 0.012) and in women (b = − 1.17 (0.31), p < 0.001). Same results were observed for DBP and PP. Negative association between BP in men was found but not in women. The small association in BP differences between heavy users and never users remains too small to adopt cannabis-blood pressure public policy in clinical practice.

## Introduction

Cannabis is the main used illicit drug. There is a worldwide trend toward to legalize cannabis, thus, it remains important to better understand the health impacts associated with its regular use. Recent studies have shown growing evidence in better cardiometabolic health associated with cannabis use^1–3^, whereas other studies suggested that cannabis use increases cardiovascular (CV) risks^4–7^. Nevertheless, these studies focused on limited populations leading to influence the impact of cannabis use on CV health^8,9^ and few studies have examined gender differences in cannabis use^10,11^. The use of medical cannabis is growing rapidly^12^, but on a limited knowledge regarding safety and efficacy in varied indications^13^. High values in blood pressure (BP) have been correlated with CV morbidity and mortality^14^. However, the association between BP and the use of cannabis remains inconsistent^10,15^. Nevertheless, a study has described that cannabis can lead to decrease BP due to vasodilatation along with tachycardia^16^. The cannabidiol (CBD), one of the major compound of cannabis, could reduce blood pressure^17^. CBD may have a sympathoinhibition action leading to decrease BP^17^. Old studies have focused on the relation between BP levels and cannabis use. Chronic use of cannabis was associated with decrease in BP^18–20^ leading to studies showing that endocannabinoid system could be a novel therapeutic way in hypertension treatment^21^. Moreover, a recent longitudinal studies showed a negative association with BP only in men^22^ whereas non-gender stratified cross-sectional studies showed positive association^15^. To date, the association between BP and cannabis use remains few studied in general populations. Thus, the purpose of our study was to examine gender stratified associations and interactions of the different lifetime aspects of cannabis use and BP levels among the general UK Biobank population.

## Methods

### UK Biobank population

The UK Biobank is a prospective cohort for the investigation, prevention, diagnosis and treatment of chronic diseases, such as CV diseases in adults. 502,478 Britons across 22 UK cities from the UK National Health Service Register were included between 2006 and 2010. The cohort was phenotyped and genotyped, by participants who responded to a questionnaire; a computer-assisted interview; physical and functional measures; and blood, urine, and saliva samples^23^. Data included socio-economic, behavior and lifestyle, mental health battery, clinical diagnoses and therapies, genetics, imaging and physiological biomarkers from blood and urine samples. The cohort protocol can be found in literature^24^.

### Ethical considerations

All participants provided electronic informed consent and UK Biobank received ethical approval from the North-West Multi-center Research Ethics Committee (MREC) covering the whole of UK. The study was conducted according to the guidelines of the Declaration of Helsinki, and approved by the North West—Haydock Research Ethics Committee (protocol code: 21/NW/0157, date of approval: 21 June 2021). For details: https://www.ukbiobank.ac.uk/learn-more-about-uk-biobank/about-us/ethics.

### Study population

**Figure 1:**
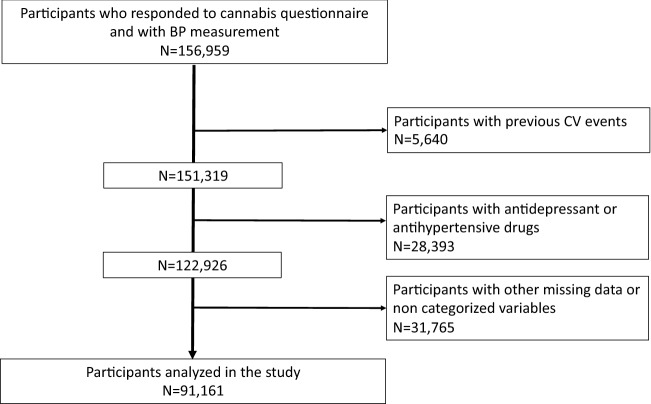
Flowchart.

156,959 volunteers of the UK Biobank who responded to the question of cannabis use and with BP measurement were recruited. Of them, we excluded 65,798 for data missing and not categorized variables and excluding participants with antihypertensive drugs, antidepressant drugs and previous CV events (Supplementary Table 1). CV events were excluded from the analyses due to the inconsistent role of cannabis in CV disorders^25^. Antidepressant drugs use were excluded due to the association between cannabis use and depression^26^. The list of antidepressant drugs was available at^27^. We therefore analyzed 91,161 volunteers (Fig. 1).

### Blood pressure measurement

Systolic and diastolic blood pressure (SBD, DBP) were measured twice at the assessment center by the use of an automated BP device (Omron 705 IT electronic blood pressure monitor; OMRON Healthcare Europe B.V. Kruisweg 577 2132 NA Hoofddorp), or manually by the use of a sphygmomanometer with an inflatable cuff in association with a stethoscope if the blood pressure device failed to measure the BP or if the largest inflatable cuff of the device did not fit around the individual’s arm^28^.

The participant was sitting in a chair for performing all the measures. The measures were carried out by nurses trained in performing BP measures^29^. Multiple available measures for one participant were averaged. The Omron 705 IT BP monitor has satisfied the Association for the Advancement of Medical Instrumentation SP10 standard and was validated by the British Hypertension Society protocol, with an overall “A” grade for both SBP and DBP^30^. 5295 participants (5.8% of the study population) had a manual sphygmomanometer BP measurement. Nevertheless, automated devices measure lower BP in comparison to manual sphygmomanometers^31^, thus, and according to previous works for UK Biobank data^32–34^, we adjusted both SBP and DBP which were measured using the automated device using algorithms:

For SBP, we performed the following algorithm:

$$SBP=3.3171+0.92019\times SBP \left(mmHg\right)+6.02468\times sex \; (male=1; \, female=0)$$

For DBP, we performed the following algorithm:

$$DBP=14.5647+0.80929\times DBP \left(mmHg\right)+2.01089\times sex \; (male=1; \, female=0)$$

Pulse pressure (PP) was calculated as = SBP − DBP.

### Cannabis use

Cannabis use was reported by self-reported questionnaire. Participants were asked about their cumulative lifetime cannabis use: ‘Have you taken cannabis (marijuana, grass, hash, ganja, blow, draw, skunk, weed, spliff, dope), even if it was a long time ago?’. Those who responded ‘no’ were classified as controls and those endorsing ‘yes’ options were classified as cannabis users. We separated these users into three groups according to categories reported in the questionnaire: those reporting initial cannabis use (‘yes, 1–2 times’, ‘yes, 3–10 times’) and continued cannabis use (‘yes, 11–100 times’: moderate users; ‘yes, more than 100 times’: heavy users). Cannabis users were asked “About how old were you when you last had cannabis?”. Cannabis user participants reporting this information and showing difference between age at inclusion and age of last cannabis use strictly inferior to 1 year were classified as “current users”, the others as “past users”. Among cannabis users, participants were asked about their cannabis frequencies use during taking: ‘Considering when you were taking cannabis most regularly, how often did you take it?’. The participants were classified into four groups, as: ‘every day’, ‘once a week or more, but not every day’, ‘once a month or more, but nor every week’, ‘less than once a month’.

### Covariates

Diabetes status was defined on either receiving anti-diabetic medication or diabetes diagnosed by a doctor or a fasting glucose concentration ≥ 7 mmol/L. Dyslipidemia was defined as having a fasting plasma total-cholesterol or triglycerides level of ≥ 6.61 mmol/L (255 mg/dL) or > 1.7 mmol/L (150 mg/dL) respectively or having statins medication^35^. Hypertension was defined as systolic blood pressure (SBP) at least 140 mm Hg and/or diastolic BP (DBP) at least 90 mm Hg^36^. Medications were characterized by the question: “Do you regularly take any of the following medications?”.

Current tobacco smokers were defined as participants who responded “yes, on most or all days” at the question “do you smoke tobacco now”. CV diseases were defined by heart attack, angina and stroke, as diagnosed by a doctor and reported in questionnaires. Body mass index was calculated as weight (in kg) divided by height^2^ (meter), and categorized as high (BMI > 30 kg/m^2^), moderate (BMI between 25 and 30 kg/m^2^) and low (less than 25 kg/m^2^). Biological parameters were detailed in the UK Biobank protocol^37^. Education level was defined in three categories high (college or university degree), intermediate (A/AS levels or equivalent, O levels/GCSEs or equivalent), and low (none of the aforementioned)^38^. Income level was defined as, high level (‘greater than *£*52,000 per year’), moderate level (between *£*18,000 and *£*51,999 per year), and low level (‘less than *£*18,000 per year)^39^. Alcohol level consumption was defined as reported in questionnaire: high level (‘daily or almost daily’), moderate level (‘three or four times a week’, or ‘once or twice a week’, or ‘one to three times a month’), and low level (‘special occasions only’ or ‘never’).

### Statistical analysis

Characteristics of the study population were described as the means with standard deviation (SD) for continuous variables. Categorical variables were described as numbers and proportions. Comparisons between groups were performed using Student’s test for continuous variables. Pearson’s χ^2^ test was performed for categorical variables. Statistical analyses were stratified on gender since blood pressure differs between men and women^40^ and a difference in cannabis consumption between gender^41^.

Firstly, this study explored the association between cumulative lifetime cannabis use and BP levels, secondly, the current or past use of cannabis consumption with BP and then, the frequency of cannabis use during taking with BP.

Associations between cannabis use and BP levels were examined with linear regression models computing regression coefficients (b) and their standard errors (SE). Firstly, gender models were adjusted for age. Secondly, gender models were adjusted for age, education, income level, alcohol consumption, tobacco habits, BMI categories, diabetes and dyslipidemia. “Never users” was considered as the referent group in the analyses. Subgroup analyses by education, income level, BMI, diabetes, dyslipidemia, alcohol and tobacco habits were performed. Interactions were examined by including simultaneously cannabis use status and one of the covariates, their interaction term and adjustment for all other covariates. Statistics were performed using SAS software (version 9.4; SAS Institute, Carry, NC). A p value < 0.05 was considered statistically significant.

### Informed consent

Written informed consent has been obtained from the patients.

## Results

Men presented higher BP levels compared to women (SBP: 137 (15) mmHg for men vs. 124 (16) mmHg for women p < 0.001 and DBP: 84 (8) mmHg for men vs. 79 (8) mmHg for women, p < 0.001), higher proportion of heavy cannabis users (4.37% vs 2.12%, p < 0.001), higher proportion of current cannabis users (3.91% vs. 2.20%, p < 0.001), higher proportion of current smokers (5.63% vs 4.29%, p < 0.001), and higher proportion of high alcohol level (28.47% vs 19.67%, p < 0.001).

**Figure 2:**
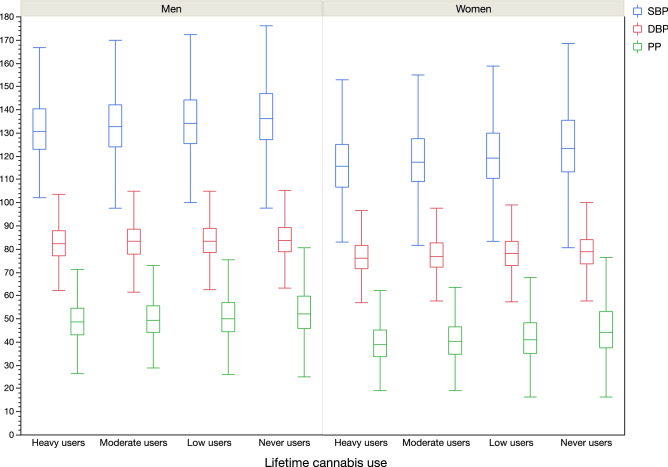
Blood pressure parameters (SBP, DBP and PP) according to gender and cannabis use groups (p < 0.001 in all subgroups).

In comparison to never users, heavy users were younger, more likely current smokers and presented higher alcohol consumption, higher levels of income and education, but lower BMI levels in both genders (Table 1). The cannabis consumption frequency was associated with the status of users. Heavy users consumed cannabis every day for 47.76% of women, and for 46.90% for men while only and 0.21% for women and 0.16% for men among low users (p < 0.001). For both SBP, DBP and PP, heavy cannabis users presented lower levels of BP compared to never users (p < 0.001) (Table 1, Fig. 2).

**Table 1:** Characteristics of the study population according to gender and cannabis use status [categorical variables with n and percentages, continuous variables with mean and standard deviation (SD)].

Men	Heavy users N = 1762 (4.4%)		Moderate users N = 2489 (6.2%)		Low users N = 7316 (18.1%)		Never users N = 28,799 (71.3%)		P value
**BMI level**									< 0.001
High	234	13.28%	374	15.03%	1231	16.83%	4927	17.11%	
Moderate	811	46.03%	1181	47.45%	3581	48.95%	14,408	50.03%	
Low	717	40.69%	934	37.53%	2504	34.23%	9464	32.86%	
**Alcohol level**									< 0.001
High	647	36.72%	868	34.87%	2355	32.19%	7624	26.47%	
Moderate	954	54.14%	1475	59.26%	4553	62.23%	18,290	63.51%	
Low	161	9.14%	146	5.87%	408	5.58%	2885	10.02%	
**Income**									< 0.001
High	765	43.42%	1351	54.28%	3704	50.63%	10,622	36.88%	
Moderate	783	44.44%	956	38.41%	3080	42.10%	15,244	52.93%	
Low	214	12.15%	182	7.31%	532	7.27%	2933	10.18%	
**Education**									< 0.001
High	1038	58.91%	1635	65.69%	4566	62.41%	13,999	48.61%	
Moderate	529	30.02%	612	24.59%	1924	26.30%	9144	31.75%	
Low	195	11.07%	242	9.72%	826	11.29%	5656	19.64%	
**Diabetes**	55	3.12%	66	2.65%	234	3.20%	1028	3.57%	0.039
**Dyslipidemia**	972	55.16%	1351	54.28%	4082	55.80%	16,238	56.38%	0.159
**Tobacco habits**									< 0.001
Current smokers	411	23.33%	233	9.36%	573	7.83%	1054	3.66%	
No current smokers	1351	76.67%	2256	90.64%	6743	92.17%	27,745	96.34%	
**Systolic BP, mmHg**	132.5	14.02	134	14.14	135.5	14.77	137.8	15.37	< 0.001
**Diastolic BP, mmHg**	82.87	7.913	83.47	8.002	83.98	7.879	84.24	7.834	< 0.001
**Hypertension**	526	29.89%	822	33.04%	2714	37.10%	12,327	42.81%	< 0.001
**Pulse pressure, mmHg**	49.63	9.276	50.51	9.462	51.52	10.26	53.58	11.12	< 0.001
**Age years**	50.24	6.825	51.28	6.933	53	7.382	56.47	7.756	< 0.001
**BMI, kg/m**^**2**^	26.24	3.743	26.47	3.701	26.78	3.791	26.83	3.682	< 0.001
**Glucose, mmol/L**	4.978	0.957	4.93	0.919	5	1.009	5.017	1.057	< 0.001
**Total cholesterol, mmol/L**	5.691	1.039	5.719	1.009	5.75	1.039	5.71	1.028	0.021
**Triglycerides, mmol/L**	1.93	1.17	1.867	1.119	1.903	1.129	1.88	1.077	0.111
**Cannabis frequency**									< 0.001
Every day	824	46.90%	89	3.23%	11	0.16%	–	–	
Once a week or more	805	45.82%	1017	41.06%	185	2.62%	–	–	
Once a month or more	107	6.09%	770	31.09%	493	6.97%	–	–	
Less than once a month	21	1.20%	610	24.63%	6384	90.26%	–	–	
**Cannabis users**									< 0.001
Current	748	42.45%	386	15.51%	446	6.10%	0	0%	
Past	1014	57.55%	2103	84.49%	6870	93.90%	0	0%	
Never	0	0%	0	0%	0	0%	28,799	100.00%	
**Time since last cannabis taking (years) for past users**	16.00	10.04	20.69	10.35	25.18	10.98	–	–	< 0.001

Women	Heavy users N = 1075 (2.1%)		Moderate users N = 2265 (4.5%)		Low users N = 7944 (15.6%)		Never users N = 39,511 (77.8%)		P value
**BMI level**									< 0.001
High	114	10.60%	280	12.36%	1073	13.51%	6347	16.06%	
Moderate	354	32.93%	647	28.57%	2610	32.85%	13,861	35.08%	
Low	607	56.47%	1338	59.07%	4261	53.64%	19,303	48.85%	
**Alcohol level**									< 0.001
High	287	26.70%	621	27.42%	1980	24.92%	7105	17.98%	
Moderate	640	59.53%	1429	63.09%	5145	64.77%	25,390	64.26%	
Low	148	13.77%	215	9.49%	819	10.31%	7016	17.76%	
**Income**									< 0.001
High	379	35.26%	977	43.13%	3517	44.27%	12,497	31.63%	
Moderate	514	47.81%	1063	46.93%	3658	46.05%	21,236	53.75%	
Low	182	16.93%	225	9.93%	769	9.68%	5778	14.62%	
**Education**									< 0.001
High	717	66.70%	1636	72.23%	5216	65.66%	18,068	45.73%	
Moderate	286	26.60%	532	23.49%	2256	28.40%	15,906	40.26%	
Low	72	6.70%	97	4.28%	472	5.94%	5537	14.01%	
**Diabetes**	24	2.23%	65	2.87%	188	2.37%	1033	2.61%	0.402
**Dyslipidemia**	384	35.72%	728	32.14%	2774	34.92%	16,626	42.08%	< 0.001
**Tobacco habits**									< 0.001
Current smokers	239	22.23%	209	9.23%	555	6.99%	1177	2.98%	
No current smokers	836	77.77%	2056	90.77%	7389	93.01%	38,334	97.02%	
**Systolic BP, mmHg**	117.4	14.82	119.3	14.63	121.2	15.36	125.4	16.61	< 0.001
**Diastolic BP, mmHg**	76.88	7.731	77.69	7.705	78.4	7.83	79.14	7.801	< 0.001
**Hypertension**	103	9.58%	258	11.40%	1136	14.31%	7983	20.22%	< 0.001
**Pulse pressure, mmHg**	40.47	9.85	41.61	9.952	42.77	10.67	46.26	12.28	< 0.001
**Age years**	50.28	6.658	50.89	6.752	51.98	7.128	55.13	7.581	< 0.001
**BMI, kg/m**^**2**^	25.05	4.016	25.13	4.459	25.5	4.41	25.96	4.541	< 0.001
**Glucose, mmol/L**	4.917	0.814	4.94	0.791	4.934	0.83	4.96	0.799	0.016
**Total cholesterol, mmol/L**	5.713	1.084	5.684	1.042	5.779	1.064	5.937	1.08	< 0.001
**Triglycerides, mmol/L**	1.396	0.837	1.304	0.722	1.323	0.722	1.417	0.761	< 0.001
**Cannabis frequency**									< 0.001
Every day	512	47.76%	81	3.62%	16	0.21%	–	–	
Once a week or more	499	46.55%	969	43.30%	202	2.66%	–	–	
Once a month or more	49	4.57%	717	32.04%	569	7.49%	–	–	
Less than once a month	12	1.12%	471	21.05%	6813	89.64%	–	–	
**Cannabis users**									< 0.001
Current	396	36.84%	320	14.13%	402	5.06%	0	0%	
Past	679	63.16%	1945	85.87%	7542	94.94%	0	0%	
Never	0	0%	0	0%	0	0%	39,511	100.00%	
**Time since last cannabis taking (years) for past users**	15.71	10.06	20.81	9.95	24.80	10.41	–	–	< 0.001

BMI: body mass index, BP: blood pressure.

Compared to never users, heavy cannabis use was associated with lower SBP in men (b = − 1.25 (0.28), p < 0.001) in age-adjusted model. Adjustment for all covariates did not affect the negative association (b = − 1.09 (0.27), p < 0.001). Same results have been observed for DBP in men with a negative association in both models (after adjustment for covariates: b = − 0.50 (0.15), p < 0.001) and for PP in men (after adjustment for covariates: b = − 0.60 (0.20), p = 0.002 (Table 2).

**Table 2:** Multiple gender linear regression models for the relationship between cannabis use and blood pressure (SBP, DBP and PP).

Men	SBP			DBP			PP	
	Beta (SE)	P value		Beta (SE)	P value		Beta (SE)	P value
**Cannabis**		< 0.001	**Cannabis**		< 0.001	**Cannabis**		< 0.001
Heavy users	− 1.09 (0.27)	< 0.001	Heavy users	− 0.50 (0.15)	< 0.001	Heavy users	− 0.60 (0.20)	0.002
Moderate users	− 0.17 (0.24)	0.362	Moderate users	− 0.09 (0.12)	0.465	Moderate users	− 0.08 (0.17)	0.649
Low users	0.35 (0.17)	0.040	Low users	0.22 (0.09)	0.010	Low users	0.12 (0.12)	0.313
Never users	Ref.		Never users	Ref.		Never users	Ref.	
**Age**	0.43 (0.01)	< 0.001	**Age**	0.02 (0.01)	< 0.001	**Age**	0.42 (0.01)	< 0.001
**Current smokers**	− 0.37 (0.16)	0.019	**Current smokers**	− 0.22 (0.08)	0.008	**Current smokers**	− 0.15 (0.11)	0.179
**Alcohol level**		< 0.001	**Alcohol level**		< 0.001	**Alcohol level**		< 0.001
High	1.88 (0.12)	< 0.001	High	1.03 (0.07)	< 0.001	High	0.85 (0.09)	< 0.001
Moderate	− 0.02 (0.11)	0.839	Moderate	− 0.02 (0.06)	0.749	Moderate	− 0.001 (0.08)	0.959
Low	Ref.		Low	Ref.		Low	Ref.	
**Income**		< 0.001	**Income**		0.025	**Income**		< 0.001
High	− 0.73 (0.12)	< 0.001	High	− 0.07 (0.06)	0.271	High	− 0.67 (0.09)	< 0.001
Moderate	0.25 (0.11)	0.023	Moderate	0.14 (0.06)	0.015	Moderate	0.11 (0.08)	0.158
Low	Ref.		Low	Ref.		Low	Ref.	
**Education**		< 0.001	**Education**		< 0.001	**Education**		< 0.001
High	− 0.80 (0.10)	< 0.001	High	− 0.33 (0.05)	< 0.001	High	− 0.47 (0.07)	< 0.001
Moderate	− 0.03 (0.11)	0.767	Moderate	− 0.01 (0.06)	0.919	Moderate	− 0.02 (0.07)	0.732
Low	Ref.		Low	Ref.		Low	Ref.	
**BMI**		< 0.001	**BMI**		< 0.001	**BMI**		< 0.001
High	3.40 (0.13)	< 0.001	High	2.89 (0.07)	< 0.001	High	0.51 (0.09)	< 0.001
Moderate	0.31 (0.09)	0.001	Moderate	0.03 (0.05)	0.517	Moderate	0.28 (0.07)	< 0.001
Low	Ref.		Low	Ref.		Low	Ref.	
**Diabetes**	0.24 (0.19)	0.223	**Diabetes**	− 0.64 (0.10)	< 0.001	**Diabetes**	0.89 (0.14)	< 0.001
**Dyslipidemia**	0.95 (0.07)	< 0.001	**Dyslipidemia**	0.60 (0.04)	< 0.001	**Dyslipidemia**	0.3( (0.05)	< 0.001

Women	SBP			DBP			PP	
	Beta (SE)	P value		Beta (SE)	P value		Beta (SE)	P value
**Cannabis**		< 0.001	**Cannabis**		< 0.001	**Cannabis**		< 0.001
Heavy users	− 1.85 (0.36)	< 0.001	Heavy users	− 0.87 (0.17)	< 0.001	Heavy users	− 0.97 (0.26)	< 0.001
Moderate users	− 0.38 (0.27)	0.153	Moderate users	− 0.09 (0.13)	0.463	Moderate users	− 0.29 (0.19)	0.139
Low users	0.38 (0.19)	0.046	Low users	0.32 (0.09)	< 0.001	Low users	0.06 (0.14)	0.686
Never users	Ref.		Never users	Ref.		Never users	Ref.	
**Age**	0.64 (0.01)	< 0.001	**Age**	0.04 (0.01)	< 0.001	**Age**	0.60 (0.01)	< 0.001
**Current smokers**	− 1.05 (0.17)	< 0.001	**Current smokers**	− 0.25 (0.08)	0.002	**Current smokers**	− 0.78 (0.09)	< 0.001
**Alcohol level**		< 0.001	**Alcohol level**		< 0.001	**Alcohol level**		< 0.001
High	1.34 (0.12)	< 0.001	High	0.89 (0.06)	< 0.001	High	0.46 (0.09)	< 0.001
Moderate	− 0.16 (0.09)	0.083	Moderate	− 0.16 (0.05)	< 0.001	Moderate	− 0.001 (0.07)	0.920
Low	Ref.		Low	Ref.		Low	Ref.	
**Income**		0.017	**Income**		0.397	**Income**		< 0.001
High	− 0.30 (0.11)	0.007	High	0.001 (0.05)	0.934	High	− 0.31 (0.08)	< 0.001
Moderate	0.11 (0.09)	0.227	Moderate	0.06 (0.04)	0.181	Moderate	0.05 (0.07)	0.447
Low	Ref.		Low	Ref.		Low	Ref.	
**Education**		< 0.001	**Education**		< 0.001	**Education**		< 0.001
High	− 1.09 (0.10)	< 0.001	High	− 0.25 (0.05)	< 0.001	High	− 0.85 (0.07)	< 0.001
Moderate	0.32 (0.10)	0.001	Moderate	0.16 (0.05)	0.001	Moderate	0.16 (0.15)	0.024
Low	Ref.		Low	Ref.		Low	Ref.	
**BMI**		< 0.001	**BMI**		< 0.001	**BMI**		< 0.001
High	3.93 (0.13)	< 0.001	High	3.29 (0.06)	< 0.001	High	0.64 (0.09)	< 0.001
Moderate	− 0.18 (0.10)	0.071	Moderate	− 0.26 (0.05)	< 0.001	Moderate	0.08 (0.07)	0.271
Low	Ref.		Low	Ref.		Low	Ref.	
**Diabetes**	0.61 (0.21)	0.004	**Diabetes**	− 0.53 (0.10)	< 0.001	**Diabetes**	1.15 (0.15)	< 0.001
**Dyslipidemia**	1.28 (0.07)	< 0.001	**Dyslipidemia**	0.68 (0.03)	< 0.001	**Dyslipidemia**	0.60 (0.05)	< 0.001

*SBP* systolic blood pressure, *DBP* diastolic blood pressure, *PP* pulse pressure, *BMI* body mass index.

In women, compared to never users, heavy cannabis use was associated with lower SBP (b = − 2.14 (0.36), p < 0.001) in age-adjusted model. Adjustment for all covariates did not affect the negative association (b = − 1.85 (0.36), p < 0.001). Same results have been observed for DBP in women with a negative association in both models (after adjustment for covariates: b = − 0.87 (0.17), p < 0.001) and for PP in women (after adjustment for covariates: b = − 0.97 (0.26), p < 0.001 (Table 2).

Table 3 showed the interaction results of the study for men. We found no interaction for men SBP between cannabis use and education (p = 0.720), income (p = 0.346), alcohol consumption (p = 0.263), BMI categories (p = 0.553). One interaction was observed with dyslipidemia (p = 0.019). The mean SBP difference for heavy cannabis users was lowered among dyslipidemia participants (b = − 0.88 (0.37), p = 0.018) compared to non-dyslipidemia participants (b = − 1.37 (0.41), p < 0.001). The interactions were significant for DBP between cannabis use and dyslipidemia (p = 0.004) with lower mean DBP difference among dyslipidemia participants. An interaction between alcohol level and cannabis use was observed (p = 0.003) for DBP among men. The mean DBP difference for heavy cannabis users was significant and higher among high alcohol consumption (b = − 0.78 (0.24), p = 0.001). The mean PP difference for heavy cannabis users was lowered among high income level (p for interaction = 0.022, with b = − 0.43 (0.27), p = 0.019).

**Table 3:** Associations of heavy cannabis users* and blood pressure levels among men, using linear regression models.

Men	SBP					DBP					PP				
Parameters	Age adjusted Estimated difference		Covariate adjusted Estimated difference		Interaction**	Age adjusted Estimated difference		Covariate adjusted Estimated difference		Interaction**	Age adjusted Estimated difference		Covariate adjusted Estimated difference		Interaction**
Current smokers	− 2.76 (0.64)	< 0.001	− 2.25 (0.63)	< 0.001	0.843	− 1.45 (0.35)	< 0.001	− 1.00 (0.40)	0.003	0.513	− 1.30 (0.43)	0.003	− 1.24 (0.44)	0.005	0.563
No current smokers	− 0.95 (0.31)	0.002	− 0.95 (0.31)	0.002		− 0.52 (0.17)	0.002	− 0.46 (0.16)	0.004		− 0.43 (0.22)	0.048	− 0.49 (0.22)	0.026	
**Alcohol**					0.263					0.003					0.813
High	− 1.67 (0.48)	< 0.001	− 1.56 (0.48)	< 0.001		− 0.97 (0.24)	< 0.001	− 0.78 (0.24)	0.001		− 0.69 (0.33)	0.039	− 0.78 (0.34)	0.020	
Moderate	− 1.10 (0.37)	0.003	− 0.77 (0.37)	0.034		− 0.57 (0.19)	0.004	− 0.31 (0.19)	0.099		− 0.53 (0.26)	0.038	− 0.45 (0.25)	0.079	
Low	− 1.61 (0.92)	0.081	− 1.18 (0.91)	0.193		− 1.19 (0.51)	0.019	− 0.73 (0.48)	0.134		− 0.42 (0.66)	0.519	− 0.46 (0.66)	0.490	
**Income**					0.346					0.998					0.022
High	− 1.05 (0.40)	0.008	− 0.86 (0.40)	0.029		− 0.56 (0.22)	0.011	− 0.42 (0.21)	0.047		− 0.50 (0.27)	0.058	− 0.43 (0.27)	0.019	
Moderate	− 1.34 (0.43)	0.002	− 1.03 (0.42)	0.014		− 0.77 (0.22)	< 0.001	− 0.50 (0.21)	0.020		− 0.56 (0.30)	0.061	− 0.53 (0.30)	0.077	
Low	− 1.93 (0.89)	0.031	− 1.73 (0.90)	0.053		− 1.00 (0.46)	0.030	− 0.81 (0.45)	0.071		− 0.93 (0.63)	0.141	− 0.91 (0.64)	0.154	
**Education**					0.720					0.565					0.897
High	− 1.22 (0.36)	< 0.001	− 1.15 (0.35)	0.001		− 0.55 (0.19)	0.005	− 0.44 (0.19)	0.019		− 0.67 (0.25)	0.006	− 0.72 (0.24)	0.004	
Moderate	− 1.09 (0.51)	0.032	− 0.81 (0.51)	0.107		− 0.83 (0.27)	0.002	− 0.57 (0.26)	0.030		− 0.26 (0.36)	0.470	− 0.24 (0.36)	0.053	
Low	− 1.81 (0.81)	0.033	− 1.55 (0.85)	0.068		− 1.05 (0.44)	0.017	− 0.66 (0.43)	0.128		− 0.76 (0.61)	0.212	− 0.89 (0.61)	0.148	
Diabetes	− 1.59 (1.58)	0.316	− 1.54 (1.61)	0.334	0.986	− 0.40 (0.83)	0.631	− 0.46(0.81)	0.576	0.357	− 1.19 (1.20)	0.319	− 1.09 (1.21)	0.369	0.662
No diabetes	− 1.25 (0.28)	< 0.001	− 1.06 (0.24)	0.472		− 0.79 (0.15)	< 0.001	− 0.49 (0.15)	< 0.001		− 0.55 (0.19)	0.005	− 0.47 (0.19)	0.004	
Dyslipidemia	− 1.13 (0.37)	0.002	− 0.88 (0.37)	0.018	0.019	− 0.72 (0.19)	< 0.001	− 0.42 (0.19)	0.029	0.004	− 0.42 (0.26)	0.113	− 0.45 (0.26)	0.085	0.298
No dyslipidemia	− 1.46 (0.41)	< 0.001	− 1.37 (0.41)	< 0.001		− 0.71 (0.22)	0.001	− 0.62 (0.23)	0.004		− 0.77 (0.28)	0.007	− 0.74 (0.29)	0.010	
**BMI**					0.553					0.159					0.913
High	− 0.15 (0.48)	0.763	− 0.18 (0.76)	0.817		− 0.14 (0.40)	0.735	− 0.07 (0.40)	0.861		− 0.03 (0.54)	0.954	− 0.11 (0.45)	0.844	
Moderate	− 0.78 (0.25)	0.002	− 1.17 (0.39)	0.003		− 0.61 (0.21)	0.003	− 0.61 (0.21)	0.003		− 0.52 (0.28)	0.067	− 0.55 (0.28)	0.049	
Low	− 0.57 (0.27)	0.037	− 1.37 (0.44)	0.002		− 0.36 (0.22)	0.108	− 0.52 (0.23)	0.021		− 0.64 (0.30)	0.035	− 0.84 (0.31)	0.006	

*SBP* systolic blood pressure, *DBP* diastolic blood pressure, *PP* pulse pressure, *BMI* body mass index.

*Referent group is never user of cannabis.

**Interaction were performed among covariate adjusted models.

Covariates adjusted estimated differences were adjusted for age, education, income level, tobacco habits, alcohol consumption, BMI categories, dyslipidemia, and diabetes, except for the stratified variables.

Table 4 showed the interaction results of the study for women. We found significant interactions for SBP between cannabis use and tobacco status (p = 0.017) and alcohol level (p = 0.039). The mean SBP difference for heavy cannabis users was higher among high current smokers (b = − 2.71 (0.76), p < 0.001) but lower among high level of alcohol (b = − 1.38 (0.71), p = 0.043). We found for women the same interactions for DBP between cannabis use and tobacco status (p = 0.031) and alcohol level (p = 0.008) in which the DBP difference for heavy cannabis users was higher among current smokers (b = − 1.49 (0.39), p < 0.001) but with no significant association with high alcohol level (only significant association with moderate alcohol level, p < 0.001). An interaction, among women, between income level and cannabis use was observed (p = 0.041) with higher DBP difference among high income level (b = − 1.25 (0.29), p < 0.001). Only on interaction between dyslipidemia and cannabis use was shown among women for PP, in which heavy cannabis users had lower mean PP difference (b = − 0.92 (0.30), p = 0.003).

**Table 4:** Associations of heavy cannabis users (Referent group is never user of cannabis) and blood pressure levels among women, using linear regression models.

WOMEN	SBP					DBP					PP				
Parameters	Age adjusted Estimated difference		Covariate adjusted Estimated difference		Interaction*	Age adjusted Estimated difference		Covariate adjusted Estimated difference		Interaction*	Age adjusted Estimated difference		Covariate adjusted Estimated difference		Interaction*
Current smokers	− 2.95 (0.78)	< 0.001	− 2.71 (0.76)	< 0.001	0.017	− 1.76 (0.41)	< 0.001	− 1.49 (0.39)	< 0.001	0.031	− 1.21 (0.53)	0.025	− 1.21 (0.54)	0.025	0.059
No current smokers	− 1.88 (0.41)	< 0.001	− 1.78 (0.41)	< 0.001		− 0.83 (0.21)	< 0.001	− 0.75 (0.19)	< 0.001		− 1.06 (0.29)	< 0.001	− 1.03 (0.29)	< 0.001	
**Alcohol**					0.039					0.008				< 0.001	0.407
High	− 1.77 (0.72)	0.014	− 1.38 (0.71)	0.043		0.76 (0.36)	0.035	− 0.61 (0.35)	0.083		− 1.01 (0.51)	0.049	− 0.77 (0.51)	0.132	
Moderate	− 2.16 (0.46)	< 0.001	− 2.00 (0.46)	< 0.001		− 1.06 (0.23)	< 0.001	− 0.98 (0.22)	< 0.001		− 1.09 (0.33)	< 0.001	− 1.01 (0.32)	0.002	
Low	− 2.87 (1.02)	0.005	− 2.15 (0.99)	0.031		− 1.25 (0.52)	0.017	− 0.88 (0.49)	0.072		− 1.63 (0.72)	0.025	− 1.26 (0.72)	0.081	
**Income**					0.489		< 0.001			0.041				< 0.001	0.377
High	− 0.83 (0.11)	< 0.001	− 2.61 (0.57)	< 0.001		− 1.30 (0.30)	< 0.001	− 1.25 (0.29)	< 0.001		− 1.47 (0.39)	< 0.001	− 1.36 (0.40)	< 0.001	
Moderate	0.23 (0.09)	0.019	− 1.52 (0.52)	0.004		− 0.93 (0.26)	< 0.001	− 0.80 (0.25)	0.002		− 0.98 (0.38)	0.011	− 0.72 (0.38)	0.060	
Low	Ref.		− 0.98 (0.94)	0.298		− 0.75 (0.47)	0.109	− 0.34 (0.45)	0.446		− 0.97 (0.69)	0.166	− 0.63 (0.70)	0.363	
**Education**					0.997		< 0.001			0.308				< 0.001	0.742
High	− 1.93 (0.43)	< 0.001	− 1.86 (0.43)	< 0.001		− 0.80 (0.22)	< 0.001	− 0.77 (0.22)	< 0.001		− 1.13 (0.30)	< 0.001	− 1.09 (0.31)	< 0.001	
Moderate	− 2.23 (0.71)	0.002	− 1.86 (0.70)	0.008		− 1.12 (0.35)	0.002	− 0.94 (0.34)	0.005		− 1.11 (0.51)	0.028	− 0.91 (0.51)	0.074	
Low	− 2.94 (1.48)	0.047	− 2.06 (1.46)	0.159		− 2.13 (0.72)	0.003	− 1.67 (0.69)	0.016		− 0.81 (1.09)	0.456	− 0.39 (1.09)	0.719	
Diabetes	− 0.23 (2.47)	0.924	− 0.29 (2.41)	0.902	0.639	− 1.18 (1.24)	0.342	− 1.34 (1.17)	0.250	0.295	0.95 (1.89)	0.604	1.04 (1.83)	0.567	0.711
No diabetes	− 2.18 (0.36)	< 0.001	− 1.89 (0.36)	< 0.001		− 0.99 (0.18)	< 0.001	− 0.86 (0.18)	< 0.001		− 1.19 (0.26)	< 0.001	− 1.03 (0.26)	< 0.001	
Dyslipidemia	− 2.47 (0.63)	< 0.001	− 1.86 (0.63)	0.003	0.411	− 1.07 (0.31)	< 0.001	− 0.77 (0.30)	0.011	0.648	− 1.06 (0.30)	< 0.001	− 0.92 (0.30)	0.003	0.029
No dyslipidemia	− 2.12 (0.43)	< 0.001	− 1.87 (0.43)	< 0.001		− 1.06 (0.22)	< 0.001	− 0.95 (0.22)	< 0.001		− 1.40 (0.46)	0.003	− 1.09 (0.46)	0.018	
**BMI**					0.209					0.490					0.152
High	− 2.71 (1.10)	0.014	− 2.65 (1.10)	0.016		− 1.43 (0.54)	0.008	− 1.41 (0.54)	0.009		− 1.28 (0.81)	0.116	− 1.23 (0.81)	0.129	
Moderate	− 1.47 (0.62)	0.019	− 1.21 (0.68)	0.055		− 0.84 (0.31)	0.006	− 0.79 (0.31)	0.009		− 0.62 (0.45)	0.168	− 0.41 (0.45)	0.373	
Low	− 2.12 (0.35)	< 0.001	− 2.07 (0.47)	< 0.001		− 0.78 (0.23)	< 0.001	− 0.82 (0.23)	< 0.001		− 1.35 (0.33)	< 0.001	− 1.25 (0.33)	< 0.001	

*SBP* systolic blood pressure, *DBP* diastolic blood pressure, *PP* pulse pressure, *BMI* body mass index.

*Interaction were performed among covariate adjusted models.

Covariates adjusted estimated differences were adjusted for age, education, income level, tobacco habits, alcohol consumption, BMI categories, dyslipidemia, and diabetes, except for the stratified variables.

**Figure 3:**
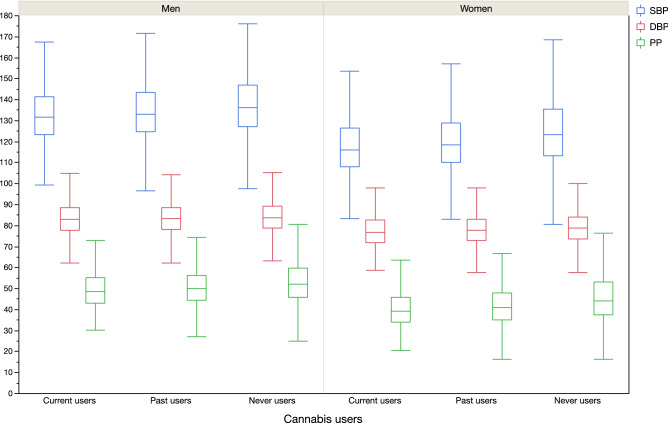
Blood pressure parameters (SBP, DBP and PP) according to gender and cannabis use groups (p < 0.001 in all subgroups).

In men, compared to never users, current cannabis use was associated with lower SBP (b = − 071 (0.25), p = 0.005) in age-adjusted model. Adjustment for all covariates did not affect the negative association (b = − 0.63 (0.25), p = 0.012). In women, compared to never users, current cannabis use was associated with lower SBP (b = − 1.35 (0.31), p < 0.001) in age-adjusted model. Adjustment for all covariates did not affect the negative association (b = − 1.17 (0.31), p < 0.001). Same results were observed for DBP and PP (Table 5, Fig. 3).

**Table 5:** Gender linear regression models for the relationship between current or past cannabis use and blood pressure (SBP, DBP and PP).

	SBP				DBP				PP			
	Age-adjusted	P value	All-covariates-adjusted*	P value	Age-adjusted	P value	All-covariates-adjusted*	P value	Age-adjusted	P value	All-covariates-adjusted*	P value
**Men**												
Cannabis												
Current users	− 0.71 (0.25)	0.005	− 0.63 (0.25)	0.012	− 0.37 (0.13)	0.006	− 0.25 (0.13)	0.044	− 0.34 (0.18)	0.045	− 0.38 (0.18)	< 0.001
Past users	− 0.13 (0.16)	0.406	− 0.08 (0.16)	0.592	− 0.01 (0.09)	0.886	− 0.01 (0.08)	0.942	− 0.15 (0.11)	0.193	− 0.08 (0.06)	0.035
Never users	Ref.		Ref.		Ref.		Ref.				Ref.	
**Women**												
Cannabis												
Current users	− 1.35 (0.31)	< 0.001	− 1.17 (0.31)	< 0.001	− 0.59 (0.16)	< 0.001	− 0.54 (0.15)	< 0.001	− 0.76 (0.22)	< 0.001	− 0.63 (0.22)	< 0.001
Past users	− 0.48 (0.19)	0.010	− 0.29 (0.18)	0.120	− 0.04 (0.09)	0.641	− 0.05 (0.09)	0.609	− 0.44 (0.13)	< 0.001	− 0.33 (0.13)	0.012
Never users	Ref.		Ref.		Ref.		Ref.		Ref.		Ref.	

*SBP* systolic blood pressure, *DBP* diastolic blood pressure, *PP* pulse pressure, *BMI* body mass index.

*model adjusted for age, education, income level, alcohol consumption, tobacco habits, BMI categories, diabetes and dyslipidemia.

In our study, we showed that no association and no interaction between frequencies of cannabis use during taking cannabis and blood pressure (SBP, DBP, PP) levels in both genders among the different groups of cannabis users (Supplementary Table 2).

### Sensitivity analysis

When considering the overall study population, heavy cannabis users showed lower SBP (b = − 1.14 (0.23), p < 0.001, with a gender interaction effect with cannabis use (p for interaction < 0.001). Same results were observed for DBP (b = − 0.68 (0.11), p < 0.001, with a p for interaction = 0.008 between gender and cannabis use), and for PP (b = − 0.74 (0.16), p < 0.001, with a p for interaction < 0.001). Same results were observed in overall study population for current cannabis use with SBP (b = − 0.73 (0.20), p < 0.001), with DBP (b = − 0.36 (0.10), p < 0.001) and with PP (b = − 0.37 (0.14), p = 0.009), with a p for interaction with gender, p < 0.001). This interaction between gender and cannabis use allows us to compare the BP effects of cannabis use between gender.

To consider the possible effect of white coat hypertension, a sensitivity analysis was performed only on the second BP measure. Spearman correlation between first and second SBP measures was very high (measure one: 131 (18) mmHg, measure two: 129 (17) mmHg, ρ = 0.895, p < 0.001), as between first and second DBP measures (measure on 81 (9) mmHg, measure two: 81 (8) mmHg, ρ = 0.869, p < 0.001). The results remained consistent for both SBP, DBP and PP when only considering second BP measurement (Supplemental Table 3).

## Discussion

The main finding of our study was that heavy cannabis use was associated with lower BP (i.e. SBP, DBP, PP) levels in both genders but in a higher manner in women (p for interaction < 0.001 in overall study population). It seems that a lifetime cannabis use was mainly associated with decrease in BP, as observed with smoking pack year^42^.

### Cannabis joint-years

As observed in our study the potential impact of time effect of cannabis use (as number of times participants took cannabis), future studies could investigated the role of cannabis with BP through a cannabis joint-years. Pack-years, for tobacco, investigates the combination of information about smoking duration and intensity^43^. A cumulative joint-years could be estimated by the equivalent of daily cannabis use for one year. At this effect, recent studies showed that the cumulative consumption of cannabis was not associate with decline in health compared to tobacco smoking^22^. Nevertheless, the cannabinoid system remains complex and it remains unclear if it can associated with worsening or improvement of metabolic health^44^.

### Cannabis use and BP

Recent studies have suggested a strongly association between cannabis use and SBP than cannabis use and DBP^45,46^. Nevertheless, the relationship between cannabis and BP remains unclear. Prospective studies have found that the increase in SBP observed for cannabis use could be mainly cofounded by a higher alcohol consumption in cannabis users^47^. In our study, we have shown an interaction between cannabis use and alcohol consumption among women, which could partly explain the higher decrease in SBP and DBP in women compared to men. In contrast, men presented an interaction between alcohol and cannabis for PP. PP is a marker of CV risk factors and arterial stiffness^48^. This relationship allows us to the possibility of cannabis use against arterial stiffness. Other recent studies have shown a better cardio-metabolic profile of cannabis users compared to non-users^44,49,50^, this could be added by the interaction observed in our study between dyslipidemia and cannabis use for SBP and DBP among men and for PP among women.

### Physiological relationship between BP and cannabis

Cannabinoids (CBs) are compounds of the Cannabis sativa plant. There are over 80 types of phytocannabinoids. The THC (Delta-9-tetrahydrocannabinol) is responsible for the psychoactive properties of cannabis^51^ while the other main phytocannainoid is the cannabidiol (CBD) which does not have psychoactive properties but interesting properties in several diseases^52^. CBD presented vasorelaxation actions in arteries^53^. Recent studies showed that CBD could reduce blood pressure^17^. This effect may be secondary to the anxiolytic properties of CBD. CBD was also responsible for endothelium-dependent vasorelaxation in mesenteric arteries. CBD could have a sympathoinhibition action leading to decrease BP^17^. Endocannabinoid system can activate CB1 receptor which increases cardiac contractile performance and reduces peripheral vascular resistance leading to lowered BP^21^. However, studies conducted in blood vessel vasomotion remain inconsistent. Even if THC may be associated with vasorelaxation, THC could enhance methoxamine-induced vasoconstriction^25^. These results may suggest that THC present different effects on vessels depending on central or peripheral properties of arteries. Nevertheless, the abrupt cessation of cannabis use was associated with increase in BP^54^.

### Alcohol consumption and cannabis use with BP levels

Prospective studies showed a possible cofounding action of alcohol consumption on the association between cannabis use and systolic BP^47^. In our study, high levels of alcohol consumption and current tobacco smoking showed associations with BP in both genders. However, interactions between heavy cannabis use with alcohol consumption and tobacco were observed only in women but not in men (except for DBP in men). This showed that the effect of heavy cannabis use on BP was higher among current women smokers than among no current women smokers. However, the effect of heavy cannabis use on BP was higher among low women alcohol users than among high women alcohol users. Alcohol consumption was considered as a main confounding factor of cannabis use impact^55^. The use of both cannabis and alcohol was the most frequent combination observed worldwide among cannabis users^47^. However, pharmacokinetic interactions have not been yet explained in depth. Nevertheless, cannabis in association with alcohol is a common administration route. A recent study has shown that high alcohol consumption was associated with increased blood THC concentration^55^. Other studies provided limited pharmacokinetics information for cannabis and alcohol due to their controlled-administration experiments^56^. Nevertheless, the mode of administration could be important in the impact of BP levels. Alcohol before smoking cannabis should not affect THC blood levels^57^. However, blood THC level was lower over 4 h after smoking with alcohol than placebo^57^ but this effect did not consider the individual cannabis use history of participants.

### Gender difference for cannabis use and BP

Gender difference effects of cannabis use have been observed in our study for BP levels with lower BP effect of heavy cannabis consumption in women compared to men. When considering all the study population, an interaction was observed between gender and cannabis use (p for interaction < 0.001, after adjustment for all covariates). Previous studies presented gender differences in frequency of cannabis use and cardiovascular response^10^. Some evidence from animal models may suggest that gonadal hormones are implicated in the cannabinoid modulation of metabolism balance and can influence cannabinoid receptor density in a gender-dependent manner^58^. Sensitivity of cannabinoid receptors were differently modulated by estrogen and testosterone probably explaining the gender differences observed. However, for the similar time duration of consumption, a previous study have shown that women smoked fewer cigarettes of cannabis compared to men and received low concentrations levels of THC in blood due to different titrate of amount cannabis smoked in response to different cannabis potencies^59^. However, higher effect has been observed among women in our study, the endocannabinoid system could mainly affect this relation even if women may have lower titrate in cannabis smoked use.

## Strengths and limitations

The major strength of the study is the large sample size of the UK Biobank cohort. The cross-sectional design can limit the causality relationship, thus reverse causation can’t be ruled out. The UK Biobank study presented a low 5.5% rate of response leading to the involvement of possible participants bias. However, given the large sample size and high internal validity, these limitations may unlikely affect the observed associations^60,61^. Moreover, the study investigation was focused on middle-aged UK participants, so our results could not be generalized to other age and ethnic populations. Nevertheless, the UK Biobank used standardized protocols to collect data, such as BP measurements. This standardization ensures replication of data collection for all participants regardless of when, where and by whom they are performed and adds external validity to our findings. Nevertheless, our study shows many limitations: socio-economic data, medical history and comorbidities were collected by self-reported questionnaires or by physician assertion during medical examination in health centers. Data of the UK Biobank were collected from 2006 to 2010. This could bias the generalization of the results to actual existing cannabis use patterns and risks. Cannabis use was self-reported by questionnaire and not by urine or blood testing. Nevertheless, the validity of self-reported cannabis use presented an overall congruence estimated at 89.8% compared to drug tests of urine specimens^62^. In our study, no data on the frequency of cannabis use around the 30 days prior to the interview was established and hence it is difficult to distinguish whether the interrelationship of cannabis use and BP is of a short term or of a chronic nature. Moreover, no data indicated current, recent or past use and should limit the results observed. No clear data were covered for THC estimation or CBD considerations. Moreover, no data were presented for smoking method, as vaping vs oral. These lack of information should be a major limitation in this study and should be investigated in further studies.

## Conclusion

We found a negative association between BP and cannabis use in both genders but with a higher manner in women. As observed for tobacco, it would be interested to develop a pack year cannabis smoking to clearly investigated the relationship between cannabis time effect consumption (as cannabis joint-years) and BP. Nevertheless, the small association in BP differences between heavy cannabis users and never users or between current cannabis users and never users remain too small to adopt cannabis-blood pressure policy in clinical practice. Longitudinal studies are needed in general populations and then, in hypertensive patients to highlight the potential lowered BP effect of cannabis in a medical use.

## Supplementary Information

Supplementary Tables.

## Data Availability

The data that support the findings of this study are available on request from the corresponding author. The data are not publicly available due to privacy or ethical restrictions.
