# Supplementary material for: Association between cannabis use and blood pressure levels according to comorbidities and socioeconomic status
Source: Sci Rep. 2023 Feb 5;13:2069. doi: 10.1038/s41598-022-22841-6 (PMC9899770; doi:10.1038/s41598-022-22841-6)
Supplement: Supplementary file 1 — Supplementary Tables. [file 41598_2022_22841_MOESM1_ESM.docx]

**Supplemental Table 1:** Characteristics of the study population and excluded population

|  | **Excluded population** | | **Study population** | | **P value** |
| --- | --- | --- | --- | --- | --- |
|  | **N= 31,765** | | **N= 91,161** | |  |
| **Gender** |  |  |  |  | **<0.001** |
| men | 10916 | 34.36% | 40366 | 44.28% |  |
| women | 20849 | 65.64% | 50795 | 55.72% |  |
| **BMI level** |  |  |  |  | 0.051 |
| High | 5135 | 16.30% | 14580 | 15.99% |  |
| Moderate | 12698 | 40.31% | 37453 | 41.08% |  |
| Low | 13665 | 43.38% | 39128 | 42.92% |  |
| **Alcohol level** |  |  |  |  | <0.001 |
| High | 6784 | 21.43% | 21487 | 23.57% |  |
| Moderate | 19776 | 62.46% | 57876 | 63.49% |  |
| Low | 5103 | 16.12% | 11798 | 12.94% |  |
| **Income** |  |  |  |  | <0.001 |
| High | 6725 | 34.19% | 33812 | 37.09% |  |
| Moderate | 10464 | 53.20% | 46534 | 51.05% |  |
| Low | 2479 | 12.60% | 10815 | 11.86% |  |
| **Education** |  |  |  |  | <0.001 |
| High | 11351 | 44.55% | 46875 | 51.42% |  |
| Moderate | 9389 | 36.85% | 31189 | 34.21% |  |
| Low | 4742 | 18.61% | 13097 | 14.37% |  |
| **Diabetes** | 550 | 3.78% | 2693 | 2.95% | <0.001 |
| **Dyslipidemia** | 12207 | 49.53% | 43155 | 47.34% | <0.001 |
| **Tobacco habits** |  |  |  |  | 0.025 |
| Current smokers | 1447 | 4.57% | 4451 | 4.88% |  |
| No current smokers | 30219 | 95.43% | 86710 | 95.12% |  |
| Cannabis use |  |  |  |  | <0.001 |
| Heavy | 688 | 2.17% | 2837 | 3.11% |  |
| Moderate | 1186 | 3.73% | 4754 | 5.21% |  |
| Low | 4136 | 13.02% | 15260 | 16.74% |  |
| Never | 25755 | 81.08% | 68310 | 74.93% |  |
| **Cannabis frequency** |  |  |  |  | 0.871 |
| Every day | 402 | 6.93% | 1524 | 6.86% |  |
| Once a week or more | 934 | 16.10% | 3677 | 16.55% |  |
| Once a month or more | 708 | 12.20% | 2705 | 12.18% |  |
| Less than once a month | 3759 | 64.78% | 14311 | 64.41% |  |
| **Cannabis status** |  |  |  |  | <0.001 |
| **Current users** | 709 | 2.23% | 2698 | 2.96% |  |
| **Past users** | 5301 | 16.69% | 20153 | 22.11% |  |
| **Never users** | 25755 | 81.08% | 68310 | 74.93% |  |
| **Systolic BP, mmHg** | 130 | 17 | 130 | 17 | 0.322 |
| **Diastolic BP, mmHg** | 81 | 8 | 81 | 8 | <0.001 |
| **Pulse pressure, mmHg** | 49 | 13 | 49 | 12 | 0.001 |
| **Age years** | 56.06 | 7.68 | 54.74 | 7.73 | <0.001 |
| **BMI, kg/m2** | 26.22 | 4.28 | 26.24 | 4.20 | 0.514 |
| **Glucose, mmol/L** | 5.02 | 0.93 | 4.97 | 0.91 | <0.001 |
| **Total cholesterol, mmol/L** | 5.89 | 1.06 | 5.81 | 1.06 | <0.001 |
| **Triglycerides, mmol/L** | 1.59 | 0.90 | 1.61 | 0.95 | 0.014 |

**Supplementary table 2**: Frequency of cannabis use and blood pressure associations among cannabis users according to low/moderate/heavy users and gender.

SBP: systolic blood pressure, DBP: diastolic blood pressure, PP: pulse pressure

| **MEN** | **SBP** | | | **DBP** | | | **PP** | | |
| --- | --- | --- | --- | --- | --- | --- | --- | --- | --- |
|  | **Low users** | **Moderate users** | **Heavy users** | **Low users** | **Moderate users** | **Heavy users** | **Low users** | **Moderate users** | **Heavy users** |
| **Cannabis frequency** |  |  |  |  |  |  |  |  |  |
| Every day | -1.32 (3.18), p=0.677 | -0.73 (1.15), p=0.519 | -0.41 (0.46), p=0.857 | 0.37 (1.71), p=0.121 | -0.29 (0.64), p=0.649 | -0.11 (0.49), p=0.827 | -0.96 (2.22), 0.665 | -1.03 (0.78), p=0.188 | -0.27 (0.59), p=0.653 |
| Once a week or more | -0.11 (1.32), p=0.929 | 0.82 (0.52), p=0.113 | 1.29 (0.87), p=0.142 | 0.58 (0.71), p=0.413 | 0.17 (0.29), p=0.577 | 0.83 (0.49), p=0.093 | -0.69 (0.92), p=0.449 | 0.65 (0.36), p=0.089 | 0.46 (0.59), p=0.442 |
| Once a month or more | -0.82 (1.18), p=0487 | 0.84 (0.54), p=0.125 | -0.79 (1.22), p=0.517 | -0.55 (0.63), p=0.382 | 0.24 (0.31), p=0.436 | 0.32 (0.69), p=0.638 | -0.26 (0.82), p=0.746 | 0.60 (0.38), p=0.110 | -1.11 (0.82), p= 0.176 |
| Less than once a month | Ref. | Ref. | Ref. | Ref. | Ref. | Ref. | Ref. | Ref. | Ref. |
| P for interaction | P=0.917 | P=0.579 | P=0.575 | P=0.361 | P=0.157 | P=0.203 | P=0.751 | P=0.107 | P=0.189 |
|  | | | | | | | | | |
| **WOMEN** | **SBP** | | | **DBP** | | | **PP** | | |
|  | **Low users** | **Moderate users** | **Heavy users** | **Low users** | **Moderate users** | **Heavy users** | **Low users** | **Moderate users** | **Heavy users** |
| **Cannabis frequency** |  |  |  |  |  |  |  |  |  |
| Every day | -2.89 (2.68), p=0.281 | -0.11 (1.17), p=0.928 | -2.19 (1.22), p=0.073 | 0.37 (1.39), p=0.785 | -0.62 (0.62), p=0.217 | -0.97 (0.65), p=0.132 | -2.51 (1.89), p=0.178 | -0.72 (0.81), p=0.367 | -1.21 (0.81), p=0.137 |
| Once a week or more | -1.97 (1.17), p=0.126 | -0.02 (0.54), p=0.973 | -0.94 (1.22), p=0.438 | 0.12 (0.61), p=0.832 | -0.46 (0.28), p=0.109 | -0.72 (0.65), p=0.264 | -1.92 (1.18), p=0.183 | 0.45 (0.37), p=0.234 | -0.22 (0.81), p=0.785 |
| Once a month or more | -1.12 (1.02), p=0.274 | 0.24 (0.57), p=0.671 | 2.58 (1.80), p=0.152 | -0.38 (0.53), p=0.472 | -0.06 (0.30), p=0.837 | 0.67 (0.95), p=0.482 | -0.74 (0.71), p=301 | 0.31 (0.39), p=438 | 1.91 (1.20), p=0.129 |
| Less than once a month | Ref. | Ref. | Ref. | Ref. | Ref. | Ref. | Ref. | Ref. | Ref. |
| P for interaction | P=0.152 | P=0.920 | P=0.663 | P=0.675 | P=0.656 | P=0.286 | P=0.256 | P=0.563 | P=0.185 |

All the models were adjusted for age, income, education, alcohol, dyslipidemia, diabetes, tobacco habits and BMI categories.

Supplementary Table 3 : Multiple gender linear regression models for the relationship between cannabis use and blood pressure (SBP, DBP and PP) with only the second measure of BP.

| **MEN** | **SBP** | |  | **DBP** | |  | **PP** | |
| --- | --- | --- | --- | --- | --- | --- | --- | --- |
|  | Beta (SE) | P value |  | Beta (SE) | P value |  | Beta (SE) | P value |
| **Cannabis** |  | <0.001 | **Cannabis** |  | <0.001 | **Cannabis** |  | <0.001 |
| Heavy users | -1.10 (0.28) | <0.001 | Heavy users | -0.51 (0.15) | <0.001 | Heavy users | -0.59 (0.20) | 0.003 |
| Moderate users | -0.15 (0.27) | 0.542 | Moderate users | -0.06 (0.12) | 0.654 | Moderate users | -0.09 (0.17) | 0.608 |
| Low users | 0.33 (0.17) | 0.0548 | Low users | 0.19 (0.09) | 0.031 | Low users | 0.13 (0.12) | 0.284 |
| Never users | Ref. |  | Never users | Ref. |  | Never users | Ref. |  |
| **Age** | 0.42 (0.01) | <0.001 | **Age** | 0.02 (0.01) | <0.001 | **Age** | 0.40 (0.01) | <0.001 |
| **Current smokers** | -0.21 (0.16) | 0.027 | **Current smokers** | -0.16 (0.08) | 0.048 | **Current smokers** | -0.05 (0.12) | 0.671 |
| **Alcohol level** |  | <0.001 | **Alcohol level** |  | <0.001 | **Alcohol level** |  | <0.001 |
| High | 1.85 (0.13) | <0.001 | High | 0.97 (0.07) | <0.001 | High | 0.878 (0.09) | <0.001 |
| Moderate | -0.01 (0.11) | 0.945 | Moderate | 0.01 (0.06) | 0.504 | Moderate | 0.03 (0.08) | 0.695 |
| Low | Ref. |  | Low | Ref. |  | Low | Ref. |  |
| **Income** |  | <0.001 | **Income** |  | 0.001 | **Income** |  | <0.001 |
| High | -0.81 (0.12) | <0.001 | High | -0.15 (0.07) | 0.022 | High | -0.67 (0.09) | <0.001 |
| Moderate | 0.25 (0.11) | 0.009 | Moderate | 0.16 (0.06) | 0.005 | Moderate | 0.13 (0.08) | 0.114 |
| Low | Ref. |  | Low | Ref. |  | Low | Ref. |  |
| **Education** |  | <0.001 | **Education** |  | <0.001 | **Education** |  | <0.001 |
| High | -0.86 (0.10) | <0.001 | High | -0.35 (0.06) | <0.001 | High | -0.51 (0.07) | <0.001 |
| Moderate | -0.04 (0.11) | 0.707 | Moderate | -0.01 (0.06) | 0.958 | Moderate | -0.04 (0.08) | 0.579 |
| Low | Ref. |  | Low | Ref. |  | Low | Ref. |  |
| **BMI** |  | <0.001 | **BMI** |  | <0.001 | **BMI** |  | <0.001 |
| High | 3.35 (0.13) | <0.001 | High | 2.82 (0.07) | <0.001 | High | 0.52 (0.10) | <0.001 |
| Moderate | 0.30 (0.10) | 0.002 | Moderate | 0.05 (0.05) | 0.345 | Moderate | 0.25 (0.07) | <0.001 |
| Low | Ref. |  | Low | Ref. |  | Low | Ref. |  |
| **Diabetes** | 0.21 (0.20) | 0.281 | **Diabetes** | -0.74 (0.11) | <0.001 | **Diabetes** | 0.95 (0.14) | <0.001 |
| **Dyslipidemia** | 0.92 (0.07) | <0.001 | **Dyslipidemia** | 0.62 (0.04) | <0.001 | **Dyslipidemia** | 0.32 (0.05) | <0.001 |
|  | | | | | | | | |
| **WOMEN** | **SBP** | |  | **DBP** | |  | **PP** | |
|  | Beta (SE) | P value |  | Beta (SE) | P value |  | Beta (SE) | P value |
| **Cannabis** |  | <0.001 | **Cannabis** |  | <0.001 | **Cannabis** |  | <0.001 |
| Heavy users | -1.73 (0.36) | <0.001 | Heavy users | -0.85 (0.18) | <0.001 | Heavy users | -0.88 (0.27) | <0.001 |
| Moderate users | -0.42 (0.27) | 0.115 | Moderate users | -0.05 (0.13) | 0.721 | Moderate users | -0.38 (0.20) | 0.058 |
| Low users | 0.35 (0.19) | 0.069 | Low users | 0.26 (0.09) | 0.006 | Low users | 0.09 (0.14) | 0.525 |
| Never users | Ref. |  | Never users | Ref. |  | Never users | Ref. |  |
| **Age** | 0.60 (0.01) | <0.001 | **Age** | 0.03 (0.01) | <0.001 | **Age** | 0.57 (0.01) | <0.001 |
| **Current smokers** | -0.86 (0.17) | <0.001 | **Current smokers** | -0.22 (0.08) | 0.010 | **Current smokers** | -0.64 (0.13) | <0.001 |
| **Alcohol level** |  | <0.001 | **Alcohol level** |  | <0.001 | **Alcohol level** |  | <0.001 |
| High | 1.37 (0.11) | <0.001 | High | 0.87 (0.06) | <0.001 | High | 0.50 (0.09) | <0.001 |
| Moderate | -0.15 (0.09) | 0.102 | Moderate | -0.15 (0.05) | 0.002 | Moderate | -0.001 (0.07) | 0.907 |
| Low | Ref. |  | Low | Ref. |  | Low | Ref. |  |
| **Income** |  | 0.006 | **Income** |  | 0.262 | **Income** |  | <0.001 |
| High | -0.33 (0.11) | 0.003 | High | 0.001 (0.06) | 0.947 | High | -0.33 (0.08) | <0.001 |
| Moderate | 0.15 (0.10) | 0.113 | Moderate | 0.08 (0.05) | 0.103 | Moderate | 0.07 (0.07) | 0.292 |
| Low | Ref. |  | Low | Ref. |  | Low | Ref. |  |
| **Education** |  | <0.001 | **Education** |  | <0.001 | **Education** |  | <0.001 |
| High | -1.19 (0.10) | <0.001 | High | -0.31 (0.05) | 0.003 | High | -0.87 (0.07) | <0.001 |
| Moderate | 0.31 (0.10) | 0.003 | Moderate | 0.15 (0.05) | 0.003 | Moderate | 0.15 (0.15) | 0.046 |
| Low | Ref. |  | Low | Ref. |  | Low | Ref. |  |
| **BMI** |  | <0.001 | **BMI** |  | <0.001 | **BMI** |  | <0.001 |
| High | 3.80 (0.13) | <0.001 | High | 3.12 (0.06) | <0.001 | High | 0.69 (0.09) | <0.001 |
| Moderate | -0.17 (0.10) | 0.097 | Moderate | -0.21 (0.05) | <0.001 | Moderate | 0.04 (0.07) | 0.607 |
| Low | Ref. |  | Low | Ref. |  | Low | Ref. |  |
| **Diabetes** | 0.47 (0.21) | 0.028 | **Diabetes** | -0.57 (0.11) | <0.001 | **Diabetes** | 1.04 (0.16) | <0.001 |
| **Dyslipidemia** | 1.23 (0.07) | <0.001 | **Dyslipidemia** | 0.70 (0.04) | <0.001 | **Dyslipidemia** | 0.54 (0.05) | <0.001 |

SBP: systolic blood pressure, DBP: diastolic blood pressure, PP: pulse pressure, BMI: body mass index

Supplementary Table 3 bis : Multiple gender linear regression models for the relationship between cannabis use and blood pressure (SBP, DBP and PP) with only the second measure of BP.

|  | **SBP** | | | | **DBP** | | | | **PP** | | | |
| --- | --- | --- | --- | --- | --- | --- | --- | --- | --- | --- | --- | --- |
| **MEN** | **Age-adjusted** | **P value** | **All-covariates-adjusted*** | **P value** | **Age-adjusted** | **P value** | **All-covariates-adjusted*** | **P value** | **Age-adjusted** | **P value** | **All-covariates-adjusted*** | **P value** |
| **Cannabis** |  |  |  |  |  |  |  |  |  |  |  |  |
| Current users | -0.60 (0.25) | 0.018 | -0.56 (0.25) | 0.027 | -0.37 (0.14) | 0.007 | -0.27 (0.13) | 0.042 | -0.23 (0.18) | 0.047 | -0.29 (0.17) | 0.019 |
| Pas users | -0.21 (0.16) | 0.185 | -0.14 (0.16) | 0.377 | -0.01 (0.09) | 0.941 | -0.01 (0.08) | 0.903 | -0.21 (0.11) | 0.072 | -0.13 (0.07) | 0.046 |
| Never users | Ref. |  | Ref. |  | Ref. |  | ref. |  |  |  | Ref. |  |
| **WOMEN** | **Age-adjusted** | **P value** | **All-covariates-adjusted** | **P value** | **Age-adjusted** | **P value** | **All-covariates-adjusted** | **P value** | **Age-adjusted** | **P value** | **All-covariates-adjusted** | **P value** |
| **Cannabis** |  |  |  |  |  |  |  |  |  |  |  |  |
| Current users | -1.15 (0.32) | <0.001 | -1.02 (0.31) | 0.001 | -0.47 (0.16) | 0.003 | -0.43 (0.15) | 0.005 | -0.68 (0.23) | 0.003 | -0.59 (0.23) | 0.010 |
| Pas users | -0.58 (0.19) | 0.002 | -0.35 (0.18) | 0.054 | -0.13 (0.09) | 0.161 | -0.04 (0.09) | 0.711 | -0.44 (0.14) | 0.001 | -0.32 (0.14) | 0.018 |
| Never users | Ref. |  | Ref. |  | Ref. |  | Ref. |  | Ref. |  | Ref. |  |
